# Laurdan Adopts Distinct, Phase-Specific Orientations in Lipid Membranes

**DOI:** 10.1021/acs.jpcb.5c02384

**Published:** 2025-06-10

**Authors:** Agnieszka Lester, Hanna Orlikowska-Rzeznik, Emilia Krok, Lukasz Piatkowski

**Affiliations:** 49632 Poznan University of Technology, Faculty of Materials Engineering and Technical Physics, Institute of Physics, Piotrowo 3, Poznan 61-138, Poland

## Abstract

For over 40 years, Laurdan has been widely used as a universal fluorescent probe for the study of lipid membranes. However, recent molecular dynamics simulations have uncovered previously unknown properties of Laurdan, revealing that it can adopt distinct conformations within the lipid bilayer, thereby influencing its molecular orientation. Despite these findings, experimental and quantitative validation has been lacking. Here, we present the first experimental study of the orientation of Laurdan in a phase-separated supported lipid bilayer, directly linking its spatial orientation to its emission spectra in liquid-ordered (L_o_) and liquid-disordered (L_d_) phases. Using azimuthally and radially polarized excitation beams, we show that in the L_o_ phase, Laurdan molecules align more parallel to the membrane normal, whereas in the L_d_ phase, they adopt a more planar orientation within the membrane. Interestingly, the emission spectra for both excitation modes converge at shorter wavelengths, but show deviations at longer wavelengths, particularly in the L_d_ phase. By refining our understanding of the behavior of Laurdan in lipid membranes, this study underlines the critical role of the molecular orientation of the dye in fluorescence-based membrane studies and highlights the need for orientation-sensitive analysis in biophysical investigations.

## Graphical abstract

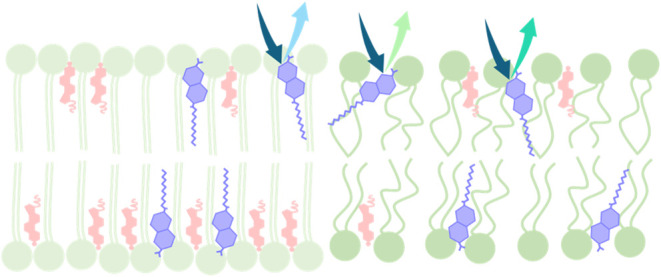

## 1 Introduction

Fluorescence microscopy is a widely used technique to determine the structure and dynamics of native and artificial cell membranes.^1,2^ One of the fundamental processes, relevant to the functions performed by the cell membrane, is the separation of membrane components and the formation of phases with distinct micro- and nanoscopic patterns.^3,4^ Two main types of fluorescent dyes are typically used to visualize phase separation in lipid membranes. The first type, phase-specific probes, selectively embed in either the liquid-ordered (L_o_) or liquid-disordered (L_d_) phase. The second type, environment-sensitive probes, partition into both lipid phases but their photophysical properties, such as the shape of their fluorescence spectra, fluorescence intensity, or fluorescence lifetime, differ depending on the local properties of the lipid membrane.^5–8^

One of the most commonly used environment-sensitive probes is Laurdan (1-(6-(dimethylamino)­naphthalen-2-yl)­dodecan-1-one), which has been used to study different aspects of membrane heterogeneity, including lipid packing, hydration levels, and fluidity—distinct but interconnected membrane biophysical properties.^9–12^ Laurdan is unique in that its emission spectrum can be shifted by up to 50 nm, depending on the lipid phase in which it is localized.^13^ In the L_o_ phase, the maximum of the fluorescence spectrum is around 425 nm, whereas in the L_d_ phase, it is shifted to longer wavelengths (∼475 nm). The shift of the emission spectrum toward lower energies results from the dipolar relaxation of the immediate Laurdan molecular environment.^9,14–16^ Thus, the fluorescence characteristics of Laurdan reflect changes in water content and other polar moieties within the membrane, as well as their reorientation dynamics.^7,9,13,17–20^ Importantly, Laurdan is evenly distributed between the L_o_ and L_d_ phases, regardless of lipid packing, and only its spectral properties change.^15,21,22^

Recent molecular dynamics (MD) simulations have shown that Laurdan can adopt different conformations in the lipid bilayer, which differ in the relative orientation of the carboxyl tail with respect to the naphthalene core.^23^ In one conformation, the amino group aligns with the membrane-water interface, while the carbonyl group aligns parallel to the lipid tails. In the other conformation, this arrangement is reversed.^23^ The shape of each conformation varies with the membrane phase. In the lipid bilayer in a gel phase (S_o_), the first conformation remains an elongated one, while the second adopts an L-shape.^23^ In the more fluid L_d_ phase, the first conformation bends, while the second becomes more elongated.^23^ As a consequence of this conformational heterogeneity, Laurdan adopts distinct spatial orientations within the membrane. The different spatial orientations of the two conformers within the membrane lead to differences in their interactions with the surrounding lipid–water interface and consequently determine the changes in the emission spectra of the probe.^24^ Osella et al.^23^ showed that Laurdan has a range of orientations within the membrane, with distinct differences between the S_o_ and L_d_ phases. In the membrane composed of DPPC, below its transition temperature (S_o_ phase), Laurdan’s transition dipole moment (TDM) has a relatively well-defined orientation, with a narrow distribution and a small difference between the two conformers.^23^ In contrast, in the DOPC membrane at ambient temperature (L_d_ phase), the orientation is much more variable, with a wide distribution of angles.^23^ One conformer has two preferred orientations, while the other has a single, but widely distributed, maximum.^23^ Overall, the L_d_ phase allows for much greater orientational flexibility, consistent with earlier theoretical results indicating a broad distribution of Laurdan orientations in pure DOPC membranes.^25^

In a recent study by Bacalum et al.,^24^ experimental work was carried out to measure the fluorescence intensity, lifetimes and generalized polarization (GP) of Laurdan in vesicles composed of DPPC or DOPC. The angular dependence of these values suggests the presence of two conformations: an extended form with higher GP and longer lifetimes (as shown for DPPC vesicles) and a bent form with lower GP and shorter lifetimes (in DOPC vesicles).^24^ The changes in the fluorescence intensity as a function of angular position further support the existence of different conformations.^24^ This study qualitatively identifies the presence of different Laurdan conformations in the membrane without quantifying their relative distribution.

As many key insights into the properties and functions of lipid membranes are derived from the spectral analysis of Laurdan, a precise understanding of its spatial and orientational distribution within the membrane is essential. Given the intrinsic heterogeneity of lipid membranes, variations in Laurdan’s insertion depth, local lipid composition, and molecular conformation could affect its spectral response - even within the same lipid phase—potentially leading to misinterpretation of the spectral data.

In this work, we experimentally investigated the orientation of Laurdan in a phase-separated lipid bilayer using azimuthally and radially polarized excitation laser beams. We found that the average orientation of Laurdan in the lipid membrane depends on the lipid phase. In the more rigid L_o_ phase, Laurdan’s TDM aligns more parallel to the lipid normal, whereas in the L_d_ phase, it is preferentially oriented within the membrane plane. In addition, we have verified the effect of Laurdan orientation on its emission spectra, demonstrating that its spectral properties are intrinsically linked to its molecular orientation within the membrane. This relationship is critical for accurately interpreting how the local membrane environment influences Laurdan fluorescence, thereby refining its application as a probe of membrane biophysical properties.

## 2 Materials and Methods

### 2.1 Materials

Egg yolk sphingomyelin (SM), 1,2-dimyristoleoyl-*sn*-glycero-3-phosphocholine (14:1 PC) and cholesterol were purchased from Avanti Polar Lipids, Alabaster, AL. 6-dodecanoyl-*N*,*N*-dimethyl-2-naphthylamine (Laurdan), 1,2-Dioleoyl-*sn*-glycero-3- phosphoethanolamine labeled with Atto 633 (DOPE-Atto 633), sodium hydroxide (NaOH), sodium chloride (NaCl) and calcium chloride (CaCl_2_) were purchased from Merck KGaA, Darmstadt, Germany. *N*-2-Hydroxyethylpiperazine-*N*′-2-ethanesulfonic acid (HEPES PUFFERAN) was purchased from Carl Roth GMBH + Co KG, Karlsruhe, Germany. Silicon Elite Double 22 Fast was purchased from Zhermack, Badia Polesine, Italy. Norland Optical Adhesive 68 glue was purchased from Norland Products Inc., Cranbury, NJ. Ultrapure water was produced using a water purification system from Labopol-Polwater, Kraków, Poland. All the materials and reagents were used without further purification.

### 2.2 Vesicles Preparation

Supported lipid bilayers (SLBs) were prepared by the vesicle deposition method on the solid support. 14:1 PC, SM and cholesterol were mixed in chloroform in a molar ratio of 1:1:1 with the addition of 10 mol % or 0.1 mol % of Laurdan, alternatively 0.1 mol % of DOPE-Atto 633 was added. The reasoning behind the use of 10 and 0.1 mol % Laurdan can be found in Supplementary Note S1. The lipid-dye mixture was dried under nitrogen gas for 20 min and then placed in a vacuum chamber for 2 h to remove residual traces of organic solvent. The dried lipid mixture was then dissolved in buffer solution (10 mM HEPES and 150 mM NaCl, pH adjusted to 7.4) to obtain a lipid concentration of 10 mM. To ensure homogeneous distribution of the lipids, the suspension was subjected to 4 cycles of heating to 60 °C and vortexing, with each step performed for 1 min. The multilamellar vesicle (MLV) solution was divided into vials, containing 10 μL of lipid suspension each, and stored at −20 °C until further use.

### 2.3 SLB Preparation

To obtain small unilamellar vesicles (SUVs), 90 μL of buffer was added to 10 μL of MLVs solution, to give a final lipid concentration of 1 mM and the mixture was sonicated for 10 min. Mica was used as a substrate for lipid deposition. A drop of immersion oil was added between the mica and the microscope coverslip, and the mica was secured to the coverslip at the edges with UV-activated adhesive. A half-cut Eppendorf tube was attached to the coverslip using medical silicone to create a buffer chamber. The 100 μL of SUVs solution, 2 μL of CaCl_2_ and 0.5 mL of buffer were applied to the mica substrate. The sample was incubated for 30 min to ensure maximum surface coverage with ruptured vesicles. After incubation, the sample was washed with the same buffer used for SLB preparation by pipetting up and down to remove any unruptured vesicles. Finally, the Eppendorf tube was filled with buffer, closed with a coverslip and sealed with silicone.

### 2.4 Imaging System

To study the orientational heterogeneity of Laurdan molecules in lipid membranes, a dedicated optical setup was designed and built. A picosecond laser diode with λ = 375 nm (PicoQuant, Germany) was used as the light source. The key element was the polarization converter (Arcoptix, Switzerland), which allowed to control the polarization of the excitation beam (linear, azimuthal or radial polarization). The polarization converter consists of a liquid crystal that rotates the orientation of the input light.^26^ A 5 μm diameter precision pinhole was used to spatially filter the outgoing laser beam to obtain a clean doughnut-shaped intensity profile. The nonpolarizing cube beam splitter (*R*/*T* 50:50) was used to direct the beam into the objective (Carl Zeiss, EC Plan-Neofluar 40*x*/1.30) and the fluorescence signal to the detector (Hamamatsu Photonics, C11202–100). The fluorescence signal was filtered from the excitation light using an optical filter (Semrock, FF01–380/LP-25). The detected fluorescence signal was converted into a digital signal by the data acquisition card (National Instruments Corporation, NI USB-6363) and acquired using custom-made LabVIEW software, whose main functions include scanning of the piezo stage for imaging purposes, acquiring photon counts from the single-photon counting modules, plotting the acquired images, and saving the data. Spectral measurements were performed by coupling the fluorescence signal to a spectrograph (Kymera 328I–C, Andor) equipped with an EMCCD camera (iXon Ultra 888, Andor), which were controlled through Andor Solis software.

## 3 Results and Discussion

To experimentally determine the spatial orientation of a molecule, the interactions between the molecular transition dipole moment (TDM) and the electromagnetic field can be used. Azimuthally and radially polarized beams can be used to measure the orientation of the TDM (see Figure 1).^27^ By exciting molecules with azimuthally and radially polarized beams and analyzing their fluorescence signal, it is possible to obtain information about the average orientation of the TDM of the molecules. In the case of Laurdan molecules, the TDM is oriented between the 2-dimethylamino and the 6-carbonyl residues and it is perpendicular to the short molecular axis of the molecule.^24,28,29^ The azimuthally polarized beam has electromagnetic field components only in the focal plane (see Figure 1B). This means that when molecules are excited with this polarization, molecules with their TDM oriented toward the plane are excited with higher efficiency. A radially polarized beam has an in-plane component in the focal plane, but it also has an intense out-of-plane component. Consequently, illuminating molecules with a radially polarized beam will result in more efficient excitation of molecules with their TDM oriented more perpendicular to the membrane plane.^27,30^

**Figure 1:**
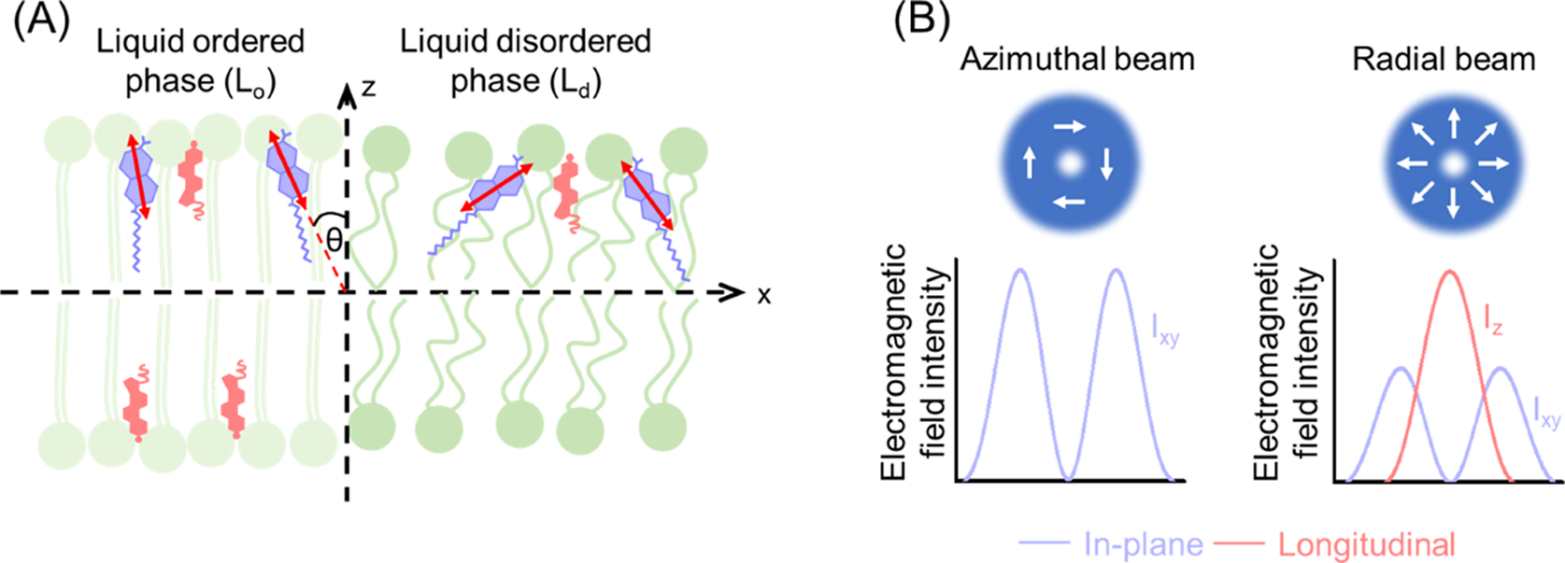
Concept of the measurements of Laurdan orientation in lipid bilayer (A) Schematic representation of the lipid phase-dependent orientation of Laurdan (blue) in a lipid bilayer with a cholesterol (coral). Red arrows indicate the direction of a transition dipole moment of the Laurdan molecule. (B) Distribution of electric field intensity at the focus of an azimuthally and radially polarized beam.

The lipid membranes, composed of 1:1:1 SM:PC:Chol, exhibit two coexisting phases—the liquid ordered (L_o_) phase, rich in sphingomyelin and cholesterol, and the liquid disordered (L_d_) phase composed mainly of unsaturated phospholipids. In the experiment, Laurdan was excited with a radially or azimuthally polarized beam generated by a liquid crystal-based polarization converter (see the Experimental section for details). Figure 2 shows the fluorescence microscopy image of the membrane excited with an azimuthally polarized beam (A) and the same area of the membrane imaged with a radially polarized beam (B). Both images show darker patches within the brighter regions. The darker patches reflect the L_o_ phase domains, which is confirmed by imaging the membrane with the additional dye DOPE-Atto633, which only partitions in the L_d_ phase (see Figure S2 in the Supporting Information). While the fluorescence intensity of the L_d_ phase is higher than that of the L_o_ phase, irrespective of the excitation polarization, the relative intensity between the phases is markedly different for the two polarizations. To highlight the differences in fluorescence intensity between the L_d_ and L_o_ phases for excitation with different polarizations, a differential image is shown in Figure 2C. Irrespective of the excitation polarization, the fluorescence intensity is similar in the L_d_ phase, but the L_o_ phase excited with an azimuthally polarized beam has a much lower intensity than when excited with a radially polarized beam. This is clearly seen in the fluorescence intensity profiles shown in Figure 2D. It should be noted that the average intensity of the excitation beam in the focal plane was the same for both polarizations. The excitation conditions between the two polarizations differ only in the distribution of the electric field in the focal plane (Figure S3). These observations clearly indicate that the average orientation of the Laurdan molecules is different in the L_o_ and L_d_ phases. In particular, the much higher fluorescence intensity of Laurdan in the L_o_ phase when excited with a radially polarized beam than with the azimuthal beam suggests that Laurdan in the L_o_ phase is oriented more perpendicular to the membrane plane than in the L_d_ phase.

**Figure 2:**
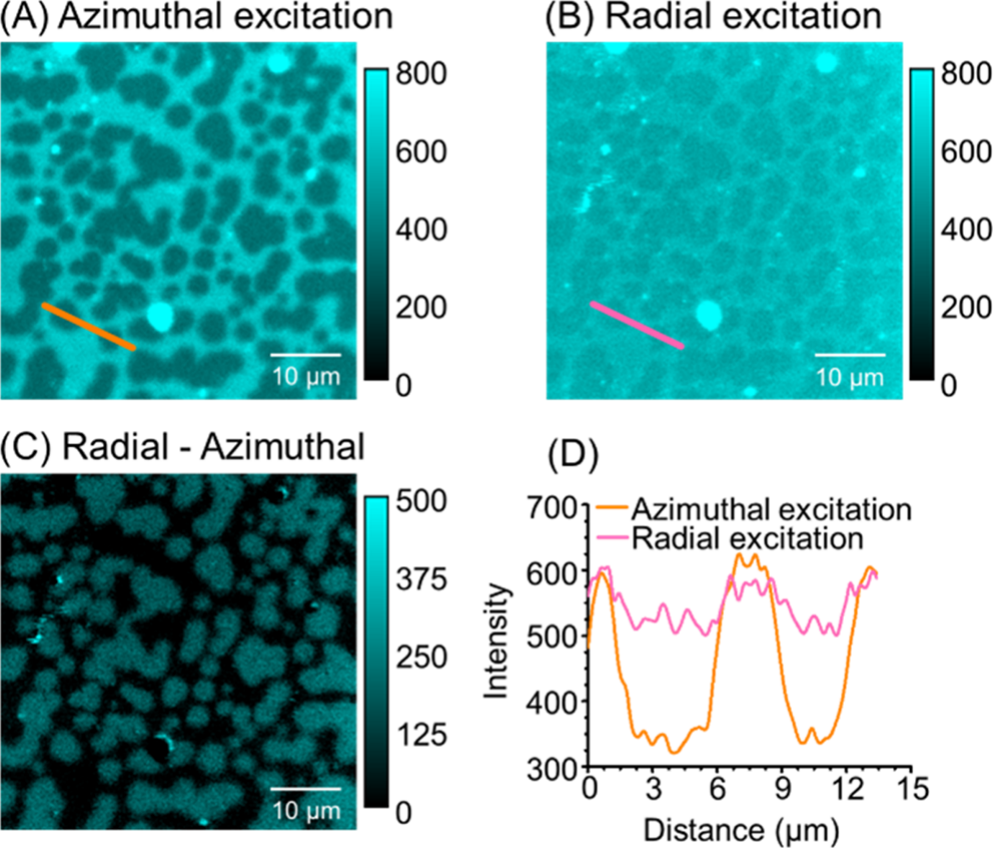
Fluorescence images of Laurdan in a lipid membrane Phase-separated SLB labeled with Laurdan, excited with (A) azimuthally polarized beam and (B) radially polarized beam. Panel (C) shows the difference between the fluorescence images from panels A and B. (D) Intensity profiles along the lines marked in the images from panels (A) and (B).

For a more quantitative picture and to determine the average orientation of Laurdan molecules in a lipid bilayer, the fluorescence intensity of the L_o_ and L_d_ phases was calculated using ImageJ/Fiji software.^31^ For each phase, the mean fluorescence intensity was determined for Laurdan excited with azimuthally (*I*_A_^Lo^, *I*_A_^Ld^) and radially (*I*_R_^Lo^, *I*_R_^Ld^) polarized beams. The ratio of fluorescence intensity obtained with azimuthal and radial beams was then calculated for each phase (*I*_A_^Lo^/*I*_R_^Lo^ and *I*_A_^Ld^/*I*_R_^Ld^). Analysis of the *I*_A_/*I*_R_ ratio gives an indication of the orientation of the molecules. Due to the fact that a radially polarized beam has electromagnetic field components both perpendicular to and within the focal plane, it is not possible to unambiguously determine the exact I_A_/I_R_ ratio at which the molecular orientation crosses the angle of 45°. However, intuitively, at lower *I*_A_/*I*_R_, the θ angle is larger and therefore the Laurdan molecules orient more in the plane, whereas higher values of *I*_A_/*I*_R_ indicate that the θ angle is smaller and the Laurdan molecules adopt a more perpendicular orientation to the membrane plane.

We have quantified the effective average orientation of Laurdan molecules in both lipid phases by comparing experimentally measured fluorescence intensity signals with theoretical calculations based on the approach of Lieb et al.,^32^ using the freely available software PMCalc. First, we computed the spatially resolved fluorescence patterns for a single molecule oriented at an arbitrary angle with respect to the focal plane and excited with azimuthally and radially polarized beams (see Figure 3A). In the calculations, we varied the angle θ, defined as the angle between the normal to the membrane surface and the TDM of the molecule (Figure 3B). The ratio of the fluorescence intensities integrated over the whole image (*I*_A_/*I*_R_) was then calculated. The dependence of the *I*_A_/*I*_R_ on the angle θ is shown in Figure 3B.

**Figure 3:**
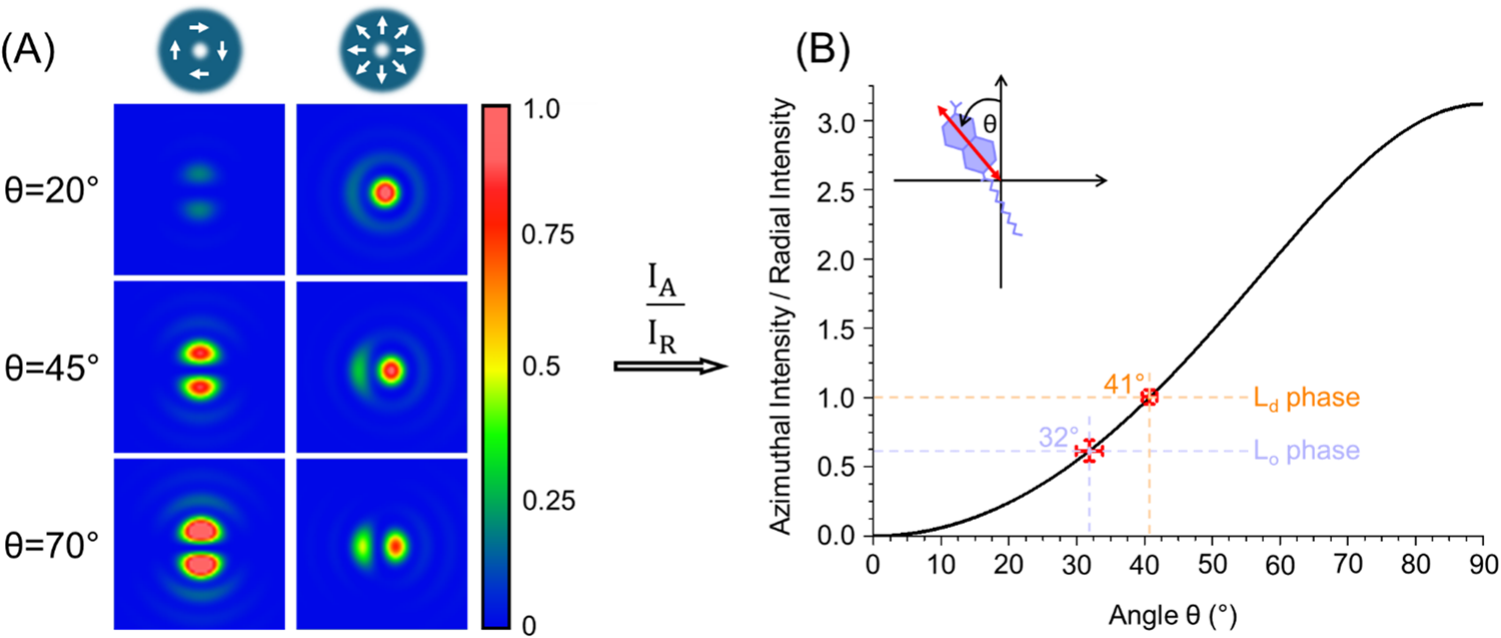
Orientation of Laurdan molecules in lipid bilayer (A) Calculated fluorescence patterns of a single Laurdan molecule excited by azimuthally and radially polarized beams with its transition dipole moment oriented at angles θ = 20°, 45° and 70° with respect to the membrane plane normal. (B) Relationship between the theoretical ratio of the fluorescence intensity of a molecule excited by azimuthally and radially polarized beams and the orientation of the molecule. The orange and purple horizontal lines indicate the experimentally determined ratio for Laurdan in the L_o_ and L_d_ phases, respectively, while the vertical lines indicate the corresponding average angle between the membrane plane and the TDM of the Laurdan population.

The calculations were performed for a single molecule, but can be extrapolated to an ensemble analysis. Given the fact that we have on average *N* = 10,000 molecules in the focal spot for 10 mol % Laurdan (for detailed calculations see Note S2 in the SI), and that this number does not change with time, we can simply assume that N is a constant scaling factor, which can simply be omitted for the ratios *I*_A_^Lo^/*I*_R_^Lo^ and *I*_A_^Ld^/*I*_R_^Ld^. The intersections between the theoretical trajectory and the experimentally determined *I*_A_/*I*_R_ values mark the average θ angle of the Laurdan molecules in the respective lipid phase. We found that in the more rigid L_o_ phase, Laurdan orientates more parallel to the membrane normal than in the L_d_ phase. The θ angle for Laurdan molecules in the L_o_ phase is approximately 32°, whereas in the L_d_ phase, it is approximately 41°.

In order to compare our results with the theoretical calculations of Osella et al.,^23^ we extracted the angle distribution data from their published plots, weighted the values by the probability of occurrence, and calculated the average for each conformation separately before averaging over both. In a membrane composed of pure DOPC, the proportions of the two conformations are reported to be roughly equal (51% and 49% for conformations I and II, respectively).^33^ To better reflect the composition and physicochemical properties of native membranes, our study included cholesterol, which is known to affect lipid packing in both the liquid disordered (L_d_) and liquid ordered (L_o_) phases. The relative amounts of the two conformers in such membranes may differ from those in single-component membranes. For consistency in our analysis, we therefore assumed that the populations were equal.

MD simulations show that, for DOPC membrane, the average angle (θ) between Laurdan’s TDM and the membrane normal is 100° in the ground state,^23,34^ and it is nearly the same for Laurdan in the first excited state (102°).^23^ In the DPPC membrane, Laurdan in the ground state adopts an orientation with an average θ angle of 68°.^23^ In relation to the experimental results, the ground state geometry is crucial due to the excitation photoselection. Nevertheless, the averaged orientations of the two Laurdan conformers in DOPC and DPPC membranes, as a function of membrane phase and for both the ground and first excited states, are summarized in Table 1 of reference.^35^

To ensure direct comparability, we have transformed the simulation-derived angles to fall within the 0–90° range, as our study reports angles within the first quadrant of the coordinate system. This transformation preserves the physical meaning, as the TDM vector projection onto the *x*-axis remains unchanged. After conversion, the average θ angle in the ground state of DOPC is 80°.

In this work we have determined the effective angle between the TDM and the normal to the lipid membrane, averaged over the conformations and their orientation distributions. In the L_d_ phase (14:1 PC and a small fraction of cholesterol), the TDM of Laurdan is oriented more toward the sample plane, whereas in the L_o_ phase (SM and a significant fraction of cholesterol), it is oriented more along the lipid tails. The same dependence was reported by Osella et al.,^23^ where in the DOPC membrane (L_d_ phase) the TDM of Laurdan is on average oriented more parallel to the membrane plane, whereas in the more rigid DPPC (S_o_ phase), the orientation of this vector is more perpendicular to the membrane normal.

The absolute values of the angles reported here are smaller compared to those derived from MD simulations (θ = 32° vs θ = 68°, for the L_o_ phase and θ = 41° vs θ = 80° for the L_d_ phase). We attribute the discrepancies to the presence of cholesterol in our system and the different conformer ratios in the membrane. It should be noted that the addition of cholesterol to the membrane significantly affects its fluidity.^9^ In phase-separated lipid bilayers, cholesterol partitions into both the L_o_ and L_d_ phases^36,37^ with a higher preference for the L_o_ phase. The fluidity of the membrane decreases with increasing cholesterol content^9^ as a consequence of the ordering of the lipid hydrocarbon chains. As a result, Laurdan molecules are forced to orient more toward the lipid tails than in a membrane without cholesterol. MD simulations show the probability of Laurdan orientation within the membrane separately for each conformer. To determine the average angle, we assumed the same population of both conformers and calculated the weighted average accordingly. However, in our study, the actual conformer ratio in each phase is not explicitly known, which may lead to discrepancies between the simulation results and our experimental observations.

It is clear that Laurdan adopts markedly different orientations in lipid membranes depending on the nature of the local environment, i.e., the lipid phase. However, the key question remains whether this is reflected in the fluorescence spectra of Laurdan. Figure 4 shows the emission spectra of Laurdan excited with azimuthally and radially polarized beams in the L_o_ and L_d_ phases. Panels A and B show the same spectra but arranged differently.

**Figure 4:**
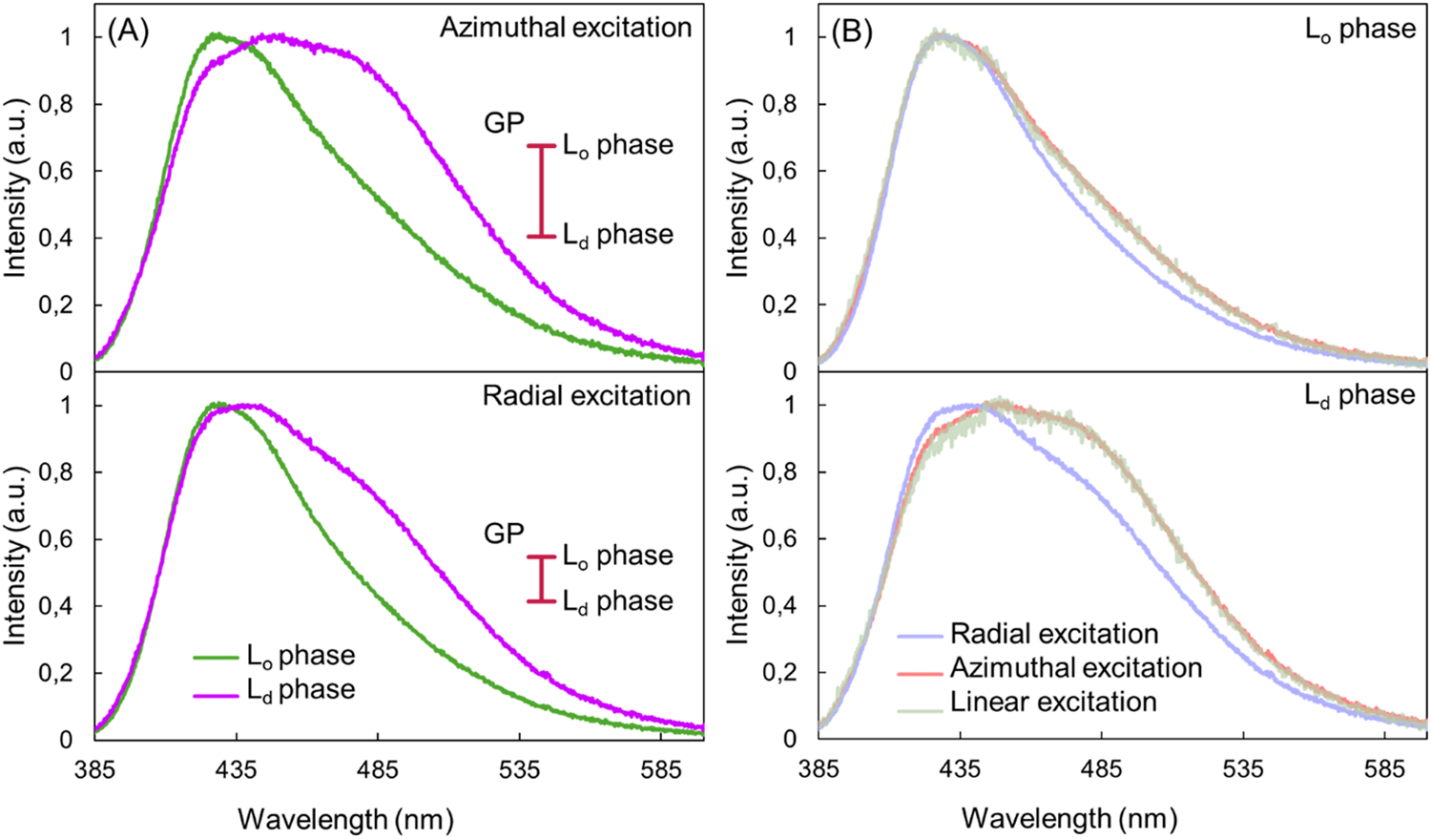
Fluorescence emission spectra of Laurdan in phase-separated SLBs excited with azimuthally, radially and linearly polarized beams (A) The differences in the emission spectra of Laurdan excited with an azimuthally polarized beam and a radially polarized beam. To the right side of the spectra, the spread of GP values is marked. For excitation with an azimuthal beam, the GP value in the L_o_ phase is 0.34, while in the L_d_ phase, it is 0.08. For excitation with a radial beam, the GP values are 0.42 and 0.19 in the L_o_ and L_d_ phases, respectively. (B) Changes in the emission spectra of Laurdan induced by excitation with different light polarizations in the L_o_ and L_d_ phases.

Figure 4A shows that the azimuthally and radially polarized beams probe different populations of Laurdan, as reflected in the different shapes of the fluorescence spectra. The difference is particularly evident for the L_d_ phase, where the Laurdan spectrum obtained with the azimuthally polarized light is significantly shifted toward longer wavelengths. It is clear that this population, favored by azimuthally polarized light, experiences a relatively high degree of dipolar relaxation, suggesting a higher exposure to water molecules and a generally unrestricted immediate environment. Conversely, a radially polarized beam interacts with both the in-plane and out-of-plane components, with greater efficiency for the out-of-plane component. This polarization effectively probes the Laurdan population, where the fluorescent moiety is oriented perpendicular to the membrane plane and experiences reduced dipolar relaxation.

To quantitatively assess these spectral variations, the generalized polarization (GP) is used as a metric to describe the fluidity of the local environment of Laurdan.^38^ In general, the higher the GP, the more rigid the membrane. GP values were calculated using the equation proposed by Parasassi et al. (see Note S3 in the SI).^39^ A larger difference in GP between L_o_ and L_d_ phases is observed for Laurdan molecules excited with an azimuthally polarized beam (see Figure S4 in SI), which is also visible as larger differences between the spectra for these phases. In addition, the GP values corresponding to the same phase are higher when excited with a radially polarized beam than with an azimuthally polarized beam (0.42 vs 0.34 for the L_o_ phase and 0.19 vs 0.08 for the L_d_ phase). Thus, when using laser light polarized in the plane of the membrane, the GP values of Laurdan are biased toward lower absolute values.

Figure 4B illustrates the differences in the spectra for each phase under different excitation beam polarizations. In the L_o_ phase, the spectra converge at shorter wavelengths and reach their maximum intensity at the same point. In contrast, in the L_d_ phase, the peak of the spectrum obtained by exciting Laurdan with a radially polarized beam is shifted toward shorter wavelengths compared to the spectrum measured with an azimuthally polarized beam. At longer wavelengths, a deviation between the spectra excited with different polarizations is observed in both phases. The differences between the spectra reflect the distribution of the angles adopted by the Laurdan molecules. In the L_o_ phase, this distribution is narrower due to the tighter packing of the lipids and the higher rigidity of this phase, whereas in the L_d_ phase, the angle distribution is broader. Panel B also shows the emission spectra of Laurdan excited with a linearly polarized beam, which are identical to the emission spectra of Laurdan excited with an azimuthally polarized beam. This is understandable because a linearly polarized beam interacts with the TDM of molecules oriented in the focal plane in a similar way to an azimuthally polarized beam. Importantly, our experimental observations are fully consistent with the molecular dynamics simulation of Baig et al.,^40^ who showed that the emission spectra of Laurdan molecules oriented more parallel to the membrane plane are shifted to longer wavelengths regardless of the lipid phase. These spectrally resolved data not only confirm that Laurdan adopts different orientations in the lipid phases, but also clearly show that Laurdan probes slightly different environments depending on its orientation.

## 4 Conclusions

In conclusion, we have demonstrated a direct measurement of the average orientation of Laurdan in a phase-separated, solid-supported lipid bilayer in both the L_o_ and L_d_ phases, and established its relationship to the fluorescence spectra of the dye. This is the first quantitative validation of computational simulations of the different orientations of Laurdan in lipid membranes, bridging the gap between theoretical modeling and experimental observations.

While the absolute values of the angles differ between simulation and experiment, likely due to variations in membrane composition, it should be emphasized that the relationship between the angles in the more rigid and more fluid phases remains consistent. Our results indicate that in a more densely packed L_o_ environment, the Laurdan molecules align more parallel to the lipid tails, whereas in the more fluid L_d_ phase, they adopt a more tilted orientation. Importantly, these different molecular orientations lead to different spectral responses, and a broader distribution of Laurdan orientation in the L_d_ phase compared to the L_o_ phase.

These findings have critical implications for biophysical studies that rely on fluorescence analysis of Laurdan. When using linearly polarized light, measurements are inherently biased toward Laurdan molecules oriented toward the membrane plane, leading to an apparent increase in membrane fluidity as indicated by the lower values of the GP parameter. This effect is particularly relevant when comparing spectral responses or GP values between different probes, which may be located at different, probe-specific depths within the membrane. By refining our understanding of the behavior of Laurdan in lipid membranes, this study highlights the importance of considering molecular orientation when interpreting the fluorescence data.

## Supplementary Material



## Data Availability

The data underlying this study are openly available in Zenodo at 10.5281/zenodo.15019219 (data for Figure 4) and 10.5281/zenodo.15019449 (data for Figure S3)

## Notes

Comparison of two images of the same sample area for DOPE-Atto 633 and Laurdan emission spectra ranges; visualization of the distribution of the electric field components in the focal spot using TetraSpeck fluorescent beads; calculation of number of Laurdan molecules in the focal spot; calculation of generalized polarization (PDF)
