# Supplementary material for: Laurdan Adopts Distinct, Phase-Specific Orientations in Lipid Membranes
Source: J Phys Chem B. 2025 Jun 10;129(25):6233–40. doi: 10.1021/acs.jpcb.5c02384 (PMC12207572; doi:10.1021/acs.jpcb.5c02384)
Supplement: Supplementary file 1 [file jp5c02384_si_001.pdf]

Supporting Information

Laurdan Adopts Distinct, Phase-Specific Orientations in Lipid Membranes

Agnieszka Lester, Hanna Orlikowska-Rzeznik, Emilia Krok, Lukasz Piatkowski**

Poznan University of Technology, Faculty of Materials Engineering and Technical Physics, Institute of Physics, Piotrowo 3, 61-138 Poznan, Poland

*agnieszka.lester@doctorate.put.poznan.pl,
*lukasz.j.piatkowski@put.poznan.pl

Supplementary Experimental Results and Supporting Information

Note 1

Measurements of Laurdan orientation in lipid bilayers were performed at two concentrations, 0.1 mol% and 10 mol%. Our analysis showed that the I_A/I_R ratio is the same (within the experimental error) for both Laurdan concentrations. In the L_o phase, the average I_A/I_R ratio was 0.6511 for Laurdan concentration of 0.1 mol% and 0.6536 at a concentration of 10 mol%. The difference in the I_A/I_R of 0.0025 translates into an angle difference of 0.09˚. In the L_d phase, the I_A/I_R ratio was 1.056 at a Laurdan concentration of 0.1 mol% and 0.9876 at a Laurdan concentration of 10 mol%, translating into an angle difference of 1.33˚. These results confirm that Laurdan concentration in the supported lipid bilayer does not significantly affect the dye's average orientation. Throughout the experiments, we have carefully monitored the structure of the lipid membranes containing 0.1 mol% and 10 mol% Laurdan and found that its presence did not affect the overall structural properties of the membrane, such as the phase separation process. Furthermore, as can be seen in the figure below, the fluorescence spectra obtained for membranes with higher and lower Laurdan concentrations are identical. This proves that Laurdan concentration has no noticeable effect on its spatial distribution within the lipid membrane, at least within the studied range of 0.1 mol% to 10 mol%.

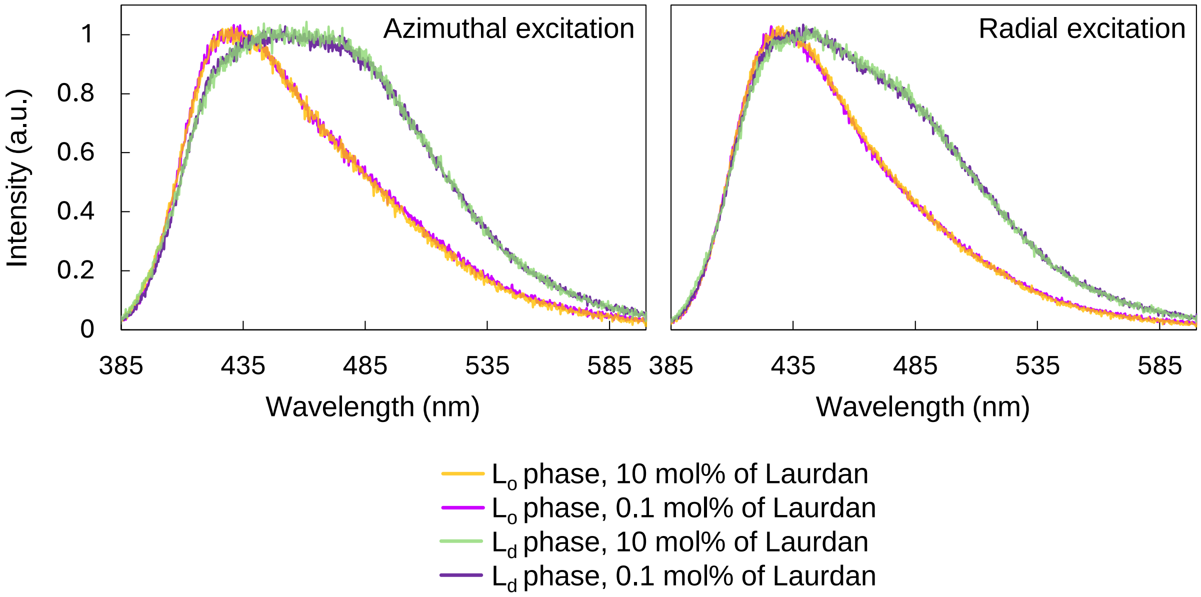

Figure S1 Comparison of normalized emission spectra at different Laurdan concentrations (10 mol% and 0.1 mol%) in the L_o and L_d phases excited with radially and azimuthally polarized beam.

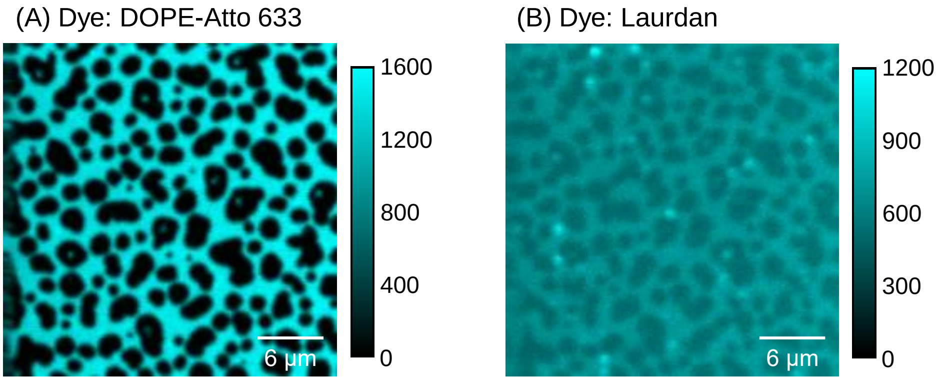

Figure S2 Comparison of two images of the same sample area, acquired using signals from different dyes. (A) The signal was collected from DOPE-Atto 633 embedded in the membrane using a 650 nm long pass filter. (B) The signal was collected exclusively from Laurdan molecules using a 475/50 nm band-pass filter. Comparison of these images confirms that Laurdan dye provides the same structural information about the membrane as DOPE-Atto 633, which partitions specifically in the L_d phase.

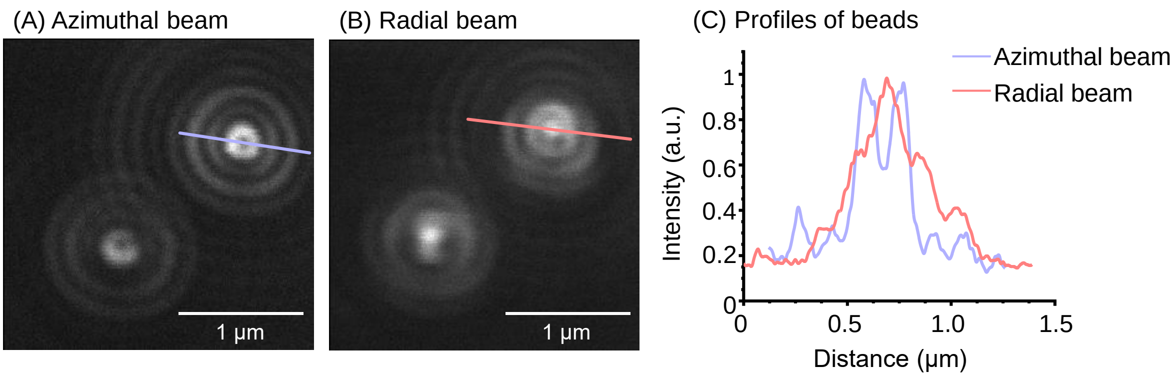

Figure S3 TetraSpeck fluorescent beads imaged using (A) azimuthally and (B) radially polarized beams to visualize the distribution of electric field components in the focal spot. (C) Fluorescence intensity profiles of the beads for both excitation beam polarizations.

Note 2

To calculate the number of Laurdan molecules in the focal spot, we assumed a lipid area of A_l = 65 Å^2[1]. The focal spot has a diameter of d_f = 300 nm, which means that there are approximately 100,000 lipid molecules in this area. For a Laurdan concentration of 10 mol%, this corresponds to approximately 10,000 Laurdan molecules in the focal spot.

Note 3

The emission spectra of Laurdan vary depending on the orientation of its fluorescent moiety within the membrane. To describe these changes, the generalized polarization (GP) was calculated using the equation proposed by Parasassi et al [2]:

$$GP = \frac{I_{440} - I_{490}}{I_{440} + I_{490}}$$

Where the I_{440} and I_{490} represent the fluorescence intensity at 440 nm and 490 nm, respectively. Intensity values were averaged over five data points around the specified wavelengths. The GP values for the L_o and L_d phases, for both excitation beam polarizations, are shown in Figure S4.

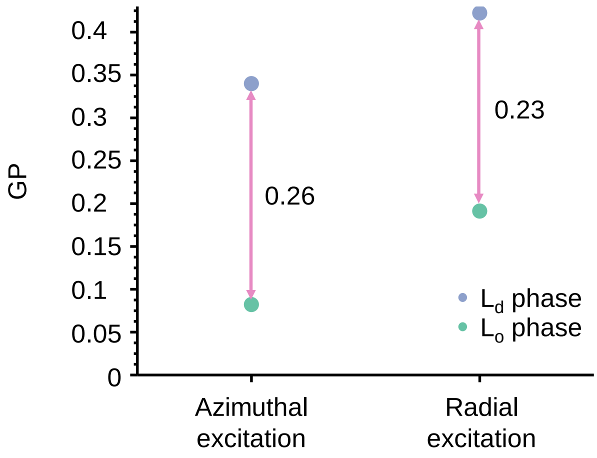

Figure S4 GP values calculated in the L_o and L_d phases for Laurdan molecules excited with azimuthally and radially polarized beams. Pink arrows and values next to them indicate the GP differences between the two phases for each excitation beam polarization.

References

[1] J. Gallová, D. Uhríková, N. Kučerka, J. Teixeira, P. Balgavý, Partial area of cholesterol in monounsaturated diacylphosphatidylcholine bilayers, Chem. Phys. Lipids 163 (2010) 765–770. https://doi.org/10.1016/j.chemphyslip.2010.08.002.

[2] T. Parasassi, G. De Stasio, A. d'Ubaldo, E. Gratton, Phase fluctuation in phospholipid membranes revealed by Laurdan fluorescence, Biophys. J. 57 (1990) 1179–1186. https://doi.org/10.1016/S0006-3495(90)82637-0.
